# History and Evolution of the Advisory Committee on Immunization Practices — United States, 1964–2014

**Published:** 2014-10-24

**Authors:** Jean Clare Smith, Alan R Hinman, Larry K. Pickering

**Affiliations:** 1 National Center for Immunization and Respiratory Diseases, CDC; 2 Center for Vaccine Equity, Task Force for Global Health, Decatur, Georgia

The Advisory Committee on Immunization Practices (ACIP) is chartered as a federal advisory committee to provide expert external advice to CDC and the Secretary of the U.S. Department of Health and Human Services (DHHS) on the use of vaccines in the civilian population of the United States. (1–3) This report summarizes the evolution of ACIP over the 50 years since its establishment in 1964 by the Surgeon General of the U.S. Public Health Service (USPHS).

During the 1940s and 1950s, USPHS relied on committees convened intermittently to address various biologics-related issues. For example, in 1955, the first effective polio vaccine was developed by Jonas Salk, at which time experts from public health, medicine, academia, the vaccine industry, and other areas were brought together on an *ad hoc* basis to deliberate on use of the vaccine. Other *ad hoc* groups were created shortly thereafter to assist the Surgeon General during the “Cutter incident,” in which cases of paralytic polio resulted from incomplete inactivation of live poliovirus in the vaccine of one manufacturer, and to review matters such as vaccine safety, effectiveness, field trials, and disease trends. By the early 1960s, with the licensure of additional new vaccines (monovalent oral poliovirus vaccine, 1961; trivalent oral poliovirus vaccine, 1963; and measles vaccine, 1963) and increased federal investment of resources in vaccines and immunization programs, it was evident that decision making on use of vaccines required a greater degree of continuity of expert technical advice rather than formation of *ad hoc* committees to address national immunization policy (4,5).

The Advisory Committee on Immunization Practice* was appointed in March 1964 by the Surgeon General of USPHS, 2 years after a proposal to establish such a committee was sent to the Surgeon General by the Secretary of the Department of Health, Education, and Welfare (DHEW†), Anthony J. Celebrezze. At the first ACIP meeting, held on May 25–26, 1964, at the Communicable Disease Center (CDC),§ the ACIP Chair, CDC Director Dr. James Goddard, presented an overview of the intended role and responsibility of the newly established committee. Other agenda topics included influenza, rubeola (measles), rubella, and smallpox, as well as the relationship of ACIP to the American Academy of Pediatrics (AAP). Minutes of that meeting included the following description of the ACIP’s responsibilities: “The Committee is charged with the responsibility of advising the Surgeon General regarding the most effective application in public health practice of specific preventive agents which may be applied in communicable disease control. Included among the agents to be considered by the Committee are inactivated and live-attenuated bacterial, rickettsial and viral agents; toxoids; anti-toxins; chemoprophylactic agents; and immune globulins. The Committee shall concern itself with immunization schedules, dosages and routes of administration and indications and contraindications for the use of these agents. The Committee shall also provide advice as to the relative priority of various population groups to whom the agents should be made available and shall advise regarding the relative merits and methods for conducting mass immunization programs. It shall also advise appropriately regarding needed programs in research.” In the 50 years since establishment of ACIP, the language of the ACIP charter has been modified, but these responsibilities remain essentially unchanged (1,6).

When ACIP was established in March 1964, it was designated as a technical advisory committee to USPHS, and comprised eight members, including the Director of CDC, who served as Chair. Members were appointed by the Secretary of DHEW, bringing expertise in public health, pediatrics, epidemiology, immunology, and preventive medicine. CDC staff contributed data on disease surveillance and epidemiology during meetings that were held at CDC’s Roybal Campus in Atlanta, Georgia, two or three times each year. In 1964, ACIP included only three liaison organizations: the AAP Committee on Infectious Diseases, the American Medical Association, and the National Advisory Committee on Immunization, Canada; and three *ex officio* members representing other federal government bodies: the Food and Drug Administration, the National Institutes of Health, and the U.S. Department of Defense.

An important change in committee procedures occurred in 1972, when the ACIP was designated a federal advisory committee. The Federal Advisory Committee Act (enacted by Public Law 92-463) is the legal foundation defining procedures for creation and operations of federal advisory committees. The law has special emphasis on open meetings, chartering, public involvement, and reporting (7). Also occurring in 1972 was a change in the reporting line of ACIP, from the Surgeon General of USPHS to the Secretary of DHEW through the Director of CDC. Two additional changes were made in 1978: the number of appointed members was increased from eight to 10 to allow participation of experts in law, ethics, and the social sciences, and it was decided that a member external to the federal government would be appointed as Chair instead of the CDC Director. The committee has continued to expand over the years and now includes 15 voting members (U.S. citizens external to the federal government), eight *ex officio* members, and 29 liaison organizations (8). Stringent measures and rigorous screening of members are used to avoid both real and perceived conflicts of interest. Vaccine manufacturers and lobbying groups do not provide financial or other support to ACIP or its members. The ACIP meets three times yearly at CDC, and may convene an emergency meeting if warranted, as was done in 2009 with the emergence of novel influenza A (H1N1).

In the 50 years since inception of ACIP, the number of vaccines included in the recommended child/adolescent immunization schedule (for persons aged 0 through 18 years) has increased from vaccines targeting six vaccine-preventable diseases to vaccines for the prevention of 16 such diseases, and the recommended immunization schedule for adults (persons aged ≥19 years) includes vaccines targeting 15 vaccine-preventable diseases (Table). The increase in the number of vaccines recommended for routine use in children and adults is reflected in the steadily increasing work load and visibility of ACIP. In 1995, the child/adolescent immunization schedule, which is updated annually, was first approved and harmonized by ACIP, the American Academy of Family Physicians (AAFP), and AAP (Figure) (9). Currently the child/adolescent immunization schedule is updated, harmonized, and approved by ACIP and professional societies including AAFP, AAP, and the American College of Obstetricians and Gynecologists (10). The recommended adult immunization schedule is updated annually and approved by ACIP, AAFP, AAP, the American College of Physicians, and the American College of Nurse Midwives (11).

Enactment of the Vaccines for Children (VFC) program in 1993 gave ACIP a new role. VFC provides an entitlement to free vaccine for all children aged 0 through 18 years who are uninsured, Medicaid eligible, American Indian/Alaska Native, or underinsured who receive vaccines at a federally qualified health center or rural health clinic; approximately 50% of U.S. children aged 0 through 18 years are VFC-eligible (12,13). If ACIP recommends that a vaccine be administered routinely to children, ACIP is then empowered to declare that the vaccine will be included in VFC.

Two additional changes have affected the work of ACIP: 1) more systematic consideration of economic analyses in development of vaccine recommendations, and 2) use of an explicit evidence based format for presentation of recommendations. Although economic data have been presented to ACIP for decades, the ACIP Charter was updated in 2004 to formally reference economic analyses. The ACIP Charter is updated and renewed by DHHS every 2 years, and the current charter (2014–2016) includes the following statement: “Committee deliberations on use of vaccines to control disease in the U.S. shall include consideration of disease epidemiology and burden of disease, vaccine efficacy and effectiveness, vaccine safety, economic analyses and implementation issues. The committee may revise or withdraw their recommendation(s) regarding a particular vaccine as new information on disease epidemiology, vaccine effectiveness or safety, economic considerations or other data becomes available.” In recent years, as the number and cost of vaccines have increased steadily, the importance of economic analyses in establishing policy for addition of new vaccines to routine immunization schedules has received increasing recognition. To ensure that economic data presented to the Committee are uniform in presentation, understandable, and of the highest quality, lead economists and the Health Economics Research Group at CDC in 2008 developed *Guidance for Health Economics Studies Presented to the ACIP*. The guidance specifically mandates technical review of any economic study that is presented to ACIP (14).

Another shift in ACIP’s approach to development of vaccine policy occurred in 2010, when the Committee voted to adopt the Grading of Recommendations Assessment, Development and Evaluation (GRADE) system to enhance transparency, continuity, and communication, and make explicit the quality of evidence reviewed (15). ACIP systematically assesses the type and quality of evidence about a vaccine’s expected health impacts and the balance of health benefits and risks, along with the values and preferences of persons affected. Evidence is grouped into four categories, with the order reflecting the level of confidence in the estimated effect of vaccination on health outcomes. Data tables used for development of ACIP vaccine recommendations are posted on the ACIP website (16).

## Discussion

The 50 years of ACIP’s progress reflects the steady increase in the number of vaccines recommended for the civilian population of the United States: from six routine childhood vaccines in 1964, to today’s 16 separate antigens that are recommended for routine use in children and adolescents, as well as the vaccines recommended for the adult population. With the passage of the Federal Advisory Committee Act in 1972, ACIP meetings became open to the public, and committee records were required to be made available to the public, thereby increasing transparency and visibility of the decision-making process. An important change was made in 1978, when the chair of the committee was appointed from among the ACIP members, none of whom is a federal government employee, thereby ensuring independence from government. Inclusion of liaison organizations representing various important professional societies or associations facilitates discussion of implementation aspects of introducing a new vaccine to the immunization program, harmonization of recommendations among stakeholders, and rapid dissemination of the recommendations back to the membership of the professional organization. The role played by ACIP in adding childhood vaccines to the VFC program has contributed to the strength of the U.S. immunization program, which has seen increases in vaccination coverage ever since the program was implemented in 1994. Although ACIP does not consider financing of vaccine programs, over the past decade the committee has regularly considered economic evaluations. Although GRADE is not applied to cost-effectiveness analyses, these considerations are taken into account by the committee, along with disease epidemiology, vaccine efficacy and effectiveness, and vaccine safety. Because of the lengthy process of data presentation and review that typically occurs over months and years before an ACIP vote is ever taken, and because of the extensive input by concerned stakeholders, ACIP immunization schedules, which summarize ACIP recommendations for routine use of vaccines in children and adults, are endorsed by medical professional organizations in the United States (10,11). In recent years, with the creation of the Bill and Melinda Gates Foundation–funded Supporting National Independent Immunization and Vaccine Advisory Committees initiative, including technical support from the World Health Organization, delegations from countries around the world have attended ACIP meetings to observe procedures followed by ACIP as they establish or enhance their own immunization advisory committees (17). Delegations from ministries of health of several countries, including Argentina, China, Japan, Mexico, Republic of Korea, Taiwan, and Turkey, have attended ACIP meetings to learn more about use of evidence in developing vaccine recommendations (18).

ACIP faces challenging issues, including optimal ways to incorporate consumer perspectives and community values. The committee also has had challenging deliberations on economic analyses in the development of vaccine recommendations and accommodating an ever increasing number of vaccines in the recommended child/adolescent immunization schedule.

## Figures and Tables

**FIGURE:**
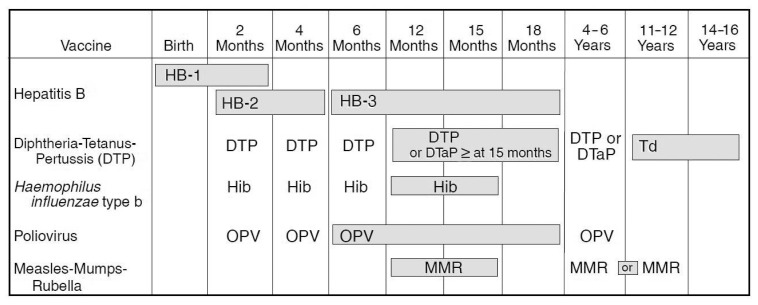
The first harmonized vaccine schedule: Recommended Childhood Immunization Schedule — United States, January 1995^*^ **Source:** CDC. Recommended childhood immunization schedule—United States, 1995;44(No. RR-5). ^*^Endorsed by Advisory Committee on Immunization Practices, American Academy of Pediatrics, and American Academy of Family Physicians.

**TABLE:** Diseases prevented by vaccines in the child/adolescent immunization schedule — United States, 1964–2014*

1964 (6 diseases)	1985 (7 diseases)	1995 (10 diseases)	2014 (16 diseases)	
Polio	Polio	Polio	Polio	Hepatitis B
Diphtheria	Diphtheria	Diphtheria	Diphtheria	Hepatitis A
Pertussis	Pertussis	Pertussis	Pertussis	Varicella
Tetanus	Tetanus	Tetanus	Tetanus	Pneumococcal
Measles	Measles	Measles	Measles	Influenza
Smallpox	Rubella	Rubella	Rubella	Meningococcal
	Mumps	Mumps	Mumps	Rotavirus
		Hib	Hib	HPV
		Hepatitis B		
		Varicella		

**Abbreviations:** Hib = *Haemophilus influenzae* type b; HPV = human papillomavirus.

* Current child/adolescent immunization schedule available at http://www.cdc.gov/vaccines/schedules/hcp/child-adolescent.html.

## Footnotes

* Renamed Advisory Committee on Immunization Practices in 1965.

† Renamed U.S. Department of Health and Human Services in 1979.

§ Renamed Centers for Disease Control in 1970, and Centers for Disease Control and Prevention in 1992.
